# Patterns of care and survival for lung cancer: Results of the European population-based high-resolution study

**DOI:** 10.3389/fepid.2023.1109853

**Published:** 2023-03-03

**Authors:** Milena Sant, Caterina Daidone, Kaire Innos, Rafael Marcos-Gragera, Katrijn Vanschoenbeek, Miguel Rodriguez Barranco, Ester Oliva Poch, Roberto Lillini, The Lung Cancer HR Study Working Group

**Affiliations:** ^1^ Analytical Epidemiology and Health Impact Unit, Department of Epidemiology and Data Science, Fondazione IRCCS Istituto Nazionale dei Tumori, Milan, Italy; ^2^ Department of Epidemiology and Biostatistics, National Institute for Health Development, Tallinn, Estonia; ^3^ Consortium for Biomedical Research in Epidemiology and Public Health (CIBERESP), Madrid, Spain; ^4^ Epidemiology Unit and Girona Cancer Registry, Oncology Coordination Plan, Department of Health, Autonomous Government of Catalonia, Catalan Institute of Oncology (ICO), Girona, Spain; ^5^ Biomedical Research Institute (IdiBGi), Girona, Spain; ^6^ Belgium Cancer Registry, Bruxelles, Belgium; ^7^ Granada Cancer Registry, Granada, Spain; ^8^ Andalusian School of Public Health (EASP), Granada, Spain; ^9^ Descriptive Epidemiology, Genetics and Cancer Prevention Group, Biomedical Research Institute (IDIBGI), Girona, Spain; ^10^ Girona Cancer Registry, Girona, Spain; ^11^ Radiation Oncology Department, Catalan Institute of Oncology Hospital Trueta, Girona, Spain

**Keywords:** lung cancer, survival, population registries, comorbidity, smoking

## Abstract

**Objectives:**

To investigate differences in lung cancer (LC) management and survival using data from European population cancer registries.

**Methods:**

We analysed 4,602 lung cancer cases diagnosed in 2010–2013, followed-up to 2019 in five countries. Multivariable logistic regression was used to calculate the Odds Ratio (OR) of surgery for stages I–II LC or chemo- or radiotherapy for stages III–IV LC. Relative survival (RS) was estimated by the actuarial method; Relative Excess Risk of death (RER), with 95% CI, was calculated by generalized linear models.

**Results:**

Diagnostic work-up was extensive for 65.9% patients (range 57%, Estonia, Portugal - 85% (Belgium). Sixty-six percent of stages I–II patients underwent surgery; compared to non-operated, their adjusted OR decreased with age and was associated with main bronchus cancer (OR vs. lobes 0.25, CI, 0.08–0.82), stage II (OR vs. stage I: 0.42, CI, 0.29–0.60), comorbidity (OR vs. absent: 0.55, CI, 0.33–0.93), country (ORs: Estonia 1.82, CI, 1.28–2.60; Belgium 0.62, CI, 0.42–0.91; Portugal 0.69, CI, 0.52–0.93).

Almost half of stages III–IV patients received chemo- or radiotherapy only; the adjusted OR vs. non receiving decreased with age and was associated with unspecified cancer topography or morphology. The adjusted five-year RER increased with age and stage and was lower for women (0.78, CI, 0.72–0.86), above the reference for main bronchus cancer (1.37, CI, 1.21–1.54) and unspecified morphology (1.17, CI, 1.05–1.30). Surgery carried the lowest mortality (RS 56.9; RER 0.13, CI, 0.11–0.15) with RER above the mean in Estonia (1.20, CI, 1.10–1.30), below it in Portugal (0.88, CI, 0.82–0.93) and Switzerland (0.91, CI, 0.84–0.99). Comorbidity (1.21, CI, 1.09–1.35) and not smoking (0.68, CI, 0.57–0.81) were associated with RER.

**Conclusions:**

The survival benefit of early diagnosis, allowing curative surgery, was evident at the population level. Screening for subjects at risk and adhesion to standard care should be incremented across the EU by funding better equipment and training health personnel.

## 1. Introduction

Lung cancer (LC) is the leading cause of cancer mortality in the world, accounting for 2.1 million new cases and 1.8 million deaths in 2018. LC constitutes 11.6% of total cancer cases and 18.4% of total cancer deaths. In males, LC is the most commonly diagnosed cancer and the leading cause of cancer death. Among women LC is the third (after breast and colorectal cancers) most commonly diagnosed cancer and the second (after breast cancer) leading cause of cancer death (1).

Despite the advent of targeted therapies and immunotherapy in non-small-cell LC (NSCLC) (2), the average five-year relative survival (RS) of European LC patients diagnosed in 2000–07 remains poor, around 13%, with figures ranging from less than 10% (UK, Lithuania, Bulgaria) to 15% or higher (Belgium, Germany, Switzerland) (3). The over-time survival improvements reported from population-based studies (4, 5) can be attributed to the higher proportions of cases diagnosed at early stages, which can benefit from curative surgery (5).

EUROCARE, the widest cancer registry based study on survival of European cancer patients, active since the early 1990s (http://www.eurocare.it/), highlighted the between-country differences in lung cancer survival for patients diagnosed in 2000–2007, were more evident for patients with localised and regional stages than for those with an advanced stage tumour at diagnosis (4); this might be ascribed to availability and access to early diagnosis and treatment facilities (3–7).

The European High-Resolution (HR) studies (http://hrstudies.it/) on samples of cancer cases archived in European population-based cancer registries (CR) collect more clinical information than is routinely provided by population CRs, according to standardised protocols, making it possible to investigate disparities in care for early stage LC that could be corrected to improve survival and reduce inequalities.

Clinical guidelines indicate surgery with curative intent should be offered to stage I–II LC patients and chemo- or radiotherapy is recommended for stage III–IV LC (8, 9); pathological confirmation is essential in deciding the appropriate treatment plan for all LC types, and chest imaging (positron emission tomography (PET), computed tomography scan (CT), spiral CT) permits precise disease staging and is essential when curative treatment is intended.

Comorbidity (particularly smoke-related, e.g., chronic obstructive pulmonary disease - COPD), smoking habits and performance status guide treatment decisions and are prognostic factors (10–13). Continued smoking in a LC patient is associated with mortality, development of a second primary, or recurrence in both non-small and small-cell LC (14).

The above indications derive from controlled clinical studies and hospital-based studies, however their impact in populations are scarcely studied. We used HR data of incident patients in 2010–2013, provided by European population CRs, to investigate:

- Adhesion to selected clinical guidelines, i.e., surgery for stage I–II LC, and chemo- or radiotherapy for stage III–IV LC, also assessing the impact of treatment on survival.
- Across-country differences in diagnostic work-up, namely the use of diagnostic examinations and the proportion of microscopically verified (MV) cases with ICD-O code subtype specification (e.g., different from not-otherwise-specified - NOS).
- Five-year survival and relative excess risk of death (RER), adjusted for clinical and demographic variables under study, also taking into account comorbidity and smoking

## 2. Materials and methods

### 2.1. Study characteristics

The HR study protocol required each participating CR to provide at least 300 cases of malignant LC [International Classification of Diseases for Oncology topography codes C34.0–9 (15), with morphologies as defined in the study protocol], diagnosed in 2010–13 and followed up to 2019 in Belgium and Spain, up to 2018 in Estonia and Switzerland and up to 2016 in Portugal (16).

The staff of each CR accessed clinical records and abstracted information on tumour stage and morphology, diagnostic work-up, treatments, smoking habits, comorbidity and life status, according to the study protocol.

Five countries with national (Belgium, Estonia) or regional coverage (Portugal, Spain, and Switzerland) contributed sufficiently complete data (i.e., most of each variable analysed for ≥70% of cases) and were included in the study. Data from regional CR were pooled in order to consider the corresponding countries as a whole.

All patients were followed up at least 5 years, according to the CR specific methods; 42 cases (less than 1%) were lost (1 in Belgium, 29 in Estonia, 4 in Portugal, 3 in Spain and 5 in Switzerland).

Comorbidity was not available for 97% of cases in Switzerland, smoking status was unavailable for 61.5% cases in Estonia (Table 1) and for 100% cases in South Portugal: these countries and this registry were therefore excluded from the relevant analyses.

**Table 1:** Number of lung cancer cases (No.) diagnosed in 2009–2013 in five European countries and distributions (%) of patients and tumour characteristic by country.

			Total cases		Belgium		Estonia		Portugal		Spain		Switzerland	
			No.	%	No.	%	No.	%	No.	%	No.	%	No.	%
All cases			4,602	*(100*.*0)*	501	*(100*.*0)*	699	*(100*.*0)*	1,433	*(100*.*0)*	1,240	*(100*.*0)*	729	*(100*.*0)*
**Age at diagnosis (years)**		15–54	585	*(12*.*7)*	45	*(9*.*0)*	63	*(9*.*0)*	217	*(15*.*2)*	172	*(13*.*9)*	88	*(12*.*1)*
		55–69	1,861	*(40*.*4)*	182	*(36*.*3)*	284	*(40*.*6)*	641	*(44*.*7)*	460	*(37*.*1)*	294	*(40*.*3)*
		≥70	2,156	*(46*.*9)*	274	*(54*.*7)*	352	*(50*.*4)*	575	*(40*.*1)*	608	*(49*.*0)*	347	*(47*.*6)*
**Sex**		Men	3,492	*(75*.*9)*	367	*(73*.*3)*	536	*(76*.*7)*	1,116	*(77*.*9)*	1,041	*(84*.*0)*	432	*(59*.*3)*
		Women	1,110	*(24*.*1)*	134	*(26*.*7)*	163	*(23*.*3)*	317	*(22*.*1)*	199	*(16*.*0)*	297	*(40*.*7)*
**Topography**		Main bronchus	359	*(7*.*8)*	33	*(6*.*6)*	42	*(6*.*0)*	54	*(3*.*8)*	169	*(13*.*6)*	61	*(8*.*4)*
		Upper lobe	2,040	*(44*.*3)*	186	*(37*.*1)*	275	*(39*.*3)*	638	*(44*.*5)*	595	*(48*.*0)*	346	*(47*.*5)*
		Middle lobe	224	*(4*.*9)*	19	*(3*.*8)*	33	*(4*.*7)*	82	*(5*.*7)*	59	*(4*.*8)*	31	*(4*.*2)*
		Lower lobe	1,132	*(24*.*6)*	107	*(21*.*4)*	161	*(23*.*0)*	356	*(24*.*9)*	311	*(25*.*1)*	197	*(27*.*0)*
		Site NOS^a^	847	*(18*.*4)*	156	*(31*.*1)*	188	*(27*.*0)*	303	*(21*.*1)*	106	*(8*.*5)*	94	*(12*.*9)*
**Morphology** ^**b**^		NSCLC	3,259	*(78*.*3)*	370	*(77*.*2)*	401	*(71*.*7)*	1,154	*(81*.*8)*	808	*(78*.*6)*	525	*(76*.*6)*
		SCLC	693	*(16*.*7)*	87	*(18*.*2)*	97	*(17*.*4)*	201	*(14*.*3)*	194	*(18*.*9)*	114	*(16*.*7)*
		NOS^a,c^	210	*(5*.*0)*	22	*(4*.*6)*	61	*(10*.*9)*	55	*(3*.*9)*	26	*(2*.*5)*	46	*(6*.*7)*
**Basis of diagnosis**		Clinical	440	*(9*.*6)*	22	*(4*.*4)*	140	*(20*.*8)*	23	*(1*.*6)*	211	*(17*.*0)*	44	*(6*.*0)*
		Microscopic	4,162	*(90*.*4)*	479	*(95*.*6)*	559	*(80*.*0)*	1,410	*(98*.*4)*	1,029	*(83*.*0)*	685	*(94*.*0)*
**TNM Stage at diagnosis**		I	496	*(10*.*8)*	59	*(11*.*8)*	87	*(12*.*4)*	140	*(9*.*8)*	138	*(11*.*1)*	72	*(9*.*9)*
		II	342	*(7*.*4)*	41	*(8*.*2)*	63	*(9*.*0)*	85	*(5*.*9)*	78	*(6*.*3)*	75	*(10*.*3)*
		III	972	*(21*.*1)*	92	*(18*.*4)*	140	*(20*.*0)*	290	*(20*.*2)*	324	*(26*.*1)*	126	*(17*.*3)*
		IV	2,371	*(51*.*5)*	225	*(44*.*9)*	323	*(46*.*2)*	827	*(57*.*7)*	664	*(53*.*5)*	332	*(45*.*5)*
		Unknown	421	*(9*.*1)*	84	*(16*.*8)*	86	*(12*.*3)*	91	*(6*.*4)*	36	*(2*.*9)*	124	*(17*.*0)*
**Treatment**	**Total Stage**	None	1,283	*(27.9)*	104	*(20.8)*	185	*(26.5)*	444	*(31.0)*	412	*(33.2)*	138	*(18*.*9)*
		Surgery	915	*(19.9)*	88	*(17.6)*	157	*(22.5)*	216	*(15.1)*	202	*(16.3)*	252	*(34*.*6)*
		Medical treatment only^d^	2,257	*(49.0)*	309	*(61.7)*	235	*(33.6)*	772	*(53.9)*	602	*(48.5)*	339	*(46*.*5)*
		Unknown	147	*(3.2)*	0	*(0.0)*	122	*(17.5)*	1	*(0.1)*	24	*(1.9)*	0	*(0*.*0)*
	**Stage I–II**	None	95	*(11.3)*	12	*(12.0)*	14	*(9.3)*	31	*(13.8)*	30	*(13.9)*	8	*(5*.*4)*
		Surgery	557	*(66.5)*	48	*(48.0)*	109	*(72.7)*	142	*(63.1)*	146	*(67.6)*	112	*(76*.*2)*
		Medical treatment only	177	*(21.1)*	40	*(40.0)*	19	*(12.7)*	51	*(22.7)*	40	*(18.5)*	27	*(18*.*4)*
		Unknown	9	*(1.1)*	0	*(0.0)*	8.0	*(5.3*	1.0	*(0.4)*	0	*(0.0)*	0	*(0*.*0)*
	**Stage III–IV**	None	1,018	*(30.5)*	64	*(20.2)*	141	*(30.5)*	355	*(31.8)*	366	*(37.0)*	92	*(20*.*1)*
		Surgery	273	*(8.2)*	18	*(5.7)*	45	*(9.7)*	65	*(5.8)*	56	*(5.7)*	86	*(18*.*8)*
		Medical treatment only	1,977	*(59.1)*	235	*(74.1)*	208	*(44.9)*	697	*(62.4)*	560	*(56.7)*	280	*(61*.*1)*
		Unknown	75	*(2.2)*	0	*(0.0)*	69	*(14.9)*	0	*(0.0)*	6	*(0.6)*	0	*(0*.*0)*
	**Unknown Stage**	None	170	*(40.4)*	28	*(33.3)*	30	*(34.9)*	58	*(63.7)*	16	*(44.4)*	38	*(30*.*6)*
		Surgery	85	*(20.2)*	21	*(25.0)*	2	*(2.3)*	8	*(8.8)*	0	*(0.0)*	54	*(43*.*5)*
		Medical treatment only	103	*(24.5)*	35	*(41.7)*	9	*(10.5)*	25	*(27.5)*	2	*(5.6)*	32	*(25*.*8)*
		Unknown	63	*(15.0)*	0	*(0.0)*	45	*(52.3)*	0	*(0.0)*	18	*(50.0)*	0	*(0*.*0)*
**Comorbidity**		No comorbidity (0 point)	1,628	*(42*.*0)*	202	*(40*.*3)*	325	*(46*.*5)*	783	*(54*.*6)*	318	*(25*.*7)*	NA	*(NA)*
		Comorbidity 1 point	830	*(21*.*4)*	124	*(24*.*7)*	127	*(18*.*2)*	302	*(21*.*1)*	277	*(22*.*3)*	NA	*(NA)*
		Low comorbidity (2 points)	675	*(17*.*4)*	69	*(13*.*8)*	134	*(19*.*2)*	196	*(13*.*7)*	276	*(22*.*3)*	NA	*(NA)*
		Severe comorbidity (>2 points)	632	*(16*.*3)*	45	*(9*.*0)*	113	*(16*.*1)*	145	*(10*.*1)*	329	*(26*.*5)*	NA	*(NA)*
		Unknown	108	*(2*.*9)*	61	*(12*.*2)*	0	*(0*.*0)*	7	*(0*.*5)*	40	*(3*.*2)*	707	*(97*.*0)*
**Smoking status**		Never	296	*(10*.*0)*	34	*(6*.*9)*	NA	*(NA)*	74	*(14*.*8)*	142	*(11*.*5)*	46	*(6*.*3)*
		Previous	1,071	*(36*.*0)*	163	*(32*.*5)*	NA	*(NA)*	205	*(41*.*0)*	502	*(40*.*5)*	201	*(27*.*6)*
		Current	1,131	*(38*.*1)*	166	*(33*.*1)*	NA	*(NA)*	162	*(32*.*4)*	453	*(36*.*5)*	350	*(48*.*0)*
		Unknown	472	*(15*.*9)*	138	*(27*.*5)*	430	*(61*.*5)*	59	*(11*.*8)*	143	*(11*.*5)*	132	*(18*.*1)*

^a^ Including Overlapping sites.

^b^ Including 1,714 adenocarcinoma, 14 sarcoma; NOS, not otherwise specified; only microscopically confirmed cases.

^c^ Only calculated for tumours with “microscopic” basis of diagnosis.

^d^ Medical treatment: chemotherapy, targeted radiotherapy, no surgery.

Most CRs provided all cases incident in one or more years of the study period. Registries covering large areas sampled cases from a defined incidence period using a randomized procedure. The majority of CRs provided one complete year of incidence (CRs of Estonia, Girona, Granada and Geneva) or a random selection of at least 500 LC cases (CR of Northern Portugal) diagnosed in 2010–13. Supplementary Table S1 shows the Total number of lung cancer cases submitted for inclusion in the HR study by country and registry, with criteria for selection of cases.

Age at diagnosis was grouped as 15–54, 55–69, 70 years or over. Tumour stage at diagnosis was coded according to the TNM 7th edition (17) and classified in stage I - IV, and unknown. When available, T and N pathological stage was preferred to the clinical stage. In the present analysis, to maximise the availability of data on stage we used the combined information from clinical and pathological stage. In case of discordance between them the most advanced stage figures were chosen. M stage was established only clinically in 92.2% of cases.

The anatomical site of the primary LC was coded according to ICD-O-3 topography and classified as main bronchus (C34.0), upper lobe (C34.1) middle lobe (C34.2) lower lobe (C34.3), overlapping (C34.8) and NOS (C34.9).

Morphology was coded according to ICD-O-3 morphology codes and grouped into NSCLC, small-cell lung cancer (SCLC), not otherwise specified carcinoma (NOS); its distribution is only presented for tumours with a “microscopic” basis for diagnosis, as defined in the (Supplementary Table S2).

Information on surgery, type of surgery and other treatments (chemotherapy, radiotherapy, or targeted treatment), was analysed as done, not done or not known whether done or not.

A score from 1 to 6 was assigned to each Charlson Comorbidity Index (CCI) item (12, 18), and the total was calculated as the sum of the scores for the 19 items. The sum was then rated as 0 point, 1 point, 2 points, >2 points and unknown. Smoking habit was grouped as current, previous, never and unknown.

For countries with information on diagnostic work-up (Belgium, Estonia, Portugal and Spain), we analysed the frequency distributions of diagnostic examinations on the lung within three months (after or before) from diagnosis and coded as done, not done and not known whether done or not.

Chest radiography (Rx), computerized tomography (CT), or pulmonary stratigraphy was classified as “conventional chest imaging”; in addition, the distributions of the following examinations were analysed individually: spiral CT, positron emission tomography (PET), magnetic resonance imaging (MRI), bronchoscopy, mediastinoscopy, endobronchial ultrasound (Table 2). All diagnostic examinations were grouped in four categories: (1) conventional chest imaging only; (2) spiral CT or PET or MRI plus any endoscopy; (3) spiral CT or PET with no endoscopy; (4) not done and unknown diagnostic examinations.

**Table 2:** Number of lung cancer cases (No.) and distributions (%) of diagnostic examinations on the lung within three months (after or before) from diagnosis in the four countries with information on diagnostic work-up.

	Total cases		Belgium		Estonia		Portugal		Spain		*χ* ^2^
	No.	%	No.	%	No.	%	No.	%	No.	%	*p*-value^a^
All cases	3,873	*(100*.*0)*	501	(100.0)	699	(100.0)	1,433	(100.0)	1,240	(100.0)	
**Conventional chest imaging** (Radiography, CT, stratigraphy)											<0.001
Done	2,635	*(68*.*0)*	451	*(90*.*0)*	478	*(64*.*4)*	646	*(45*.*1)*	1,060	*(85*.*5)*	
Not done	195	*(5*.*0)*	0	*(0*.*0)*	51	*(7*.*3)*	1	*(0*.*1)*	143	*(11*.*5)*	
Unknown	1,043	*(27*.*0)*	50	*(10*.*0)*	170	*(24*.*3)*	786	*(54*.*8)*	37	*(3*.*0)*	
**Spiral CT** ^**b**^		* *		* *	* *		* *		* *		<0.001
Done	2,877	*(74*.*3)*	474	*(94*.*6)*	534	*(76*.*4)*	707	*(49*.*3)*	1,162	*(93*.*7)*	
Not done	464	*(12*.*0)*	15	*(3*.*0)*	9	*(1*.*3)*	400	*(28*.*0)*	40	*(3*.*2)*	
Unknown	532	*(13*.*7)*	12	*(2*.*4)*	156	*(22*.*3)*	326	*(22*.*7)*	38	*(3*.*1)*	
PET^c^											
Done	1,368	*(35*.*3)*	298	*(59*.*5)*	56	*(8*.*0)*	459	*(32*.*0)*	555	*(44*.*8)*	
Not done	1,453	*(37*.*5)*	200	*(39*.*9)*	486	*(69*.*5)*	127	*(8*.*9)*	640	*(51*.*6)*	
Unknown	1,052	*(27*.*2)*	3	*(0*.*6)*	157	*(22*.*5)*	847	*(59*.*1)*	45	*(3*.*6)*	
**Magnetic resonance imaging**		* *		* *	* *		* *		* *		<0.001
Done	262	*(6*.*8)*	0	*(0*.*0)*	16	*(2*.*3)*	151	*(10*.*5)*	95	*(7*.*7)*	
Not done	1,993	*(51*.*5)*	0	*(0*.*0)*	529	*(75*.*7)*	369	*(25*.*8)*	1,095	*(88*.*3)*	
Unknown	1,618	*(41*.*8)*	501	*(100*.*0)*	154	*(22*.*0)*	913	*(63*.*7)*	50	*(4*.*0)*	
**Bronchoscopy**		* *		* *	* *		* *		* *		
Done	2,447	*(63*.*2)*	405	*(80*.*8)*	376	*(53*.*8)*	808	*(56*.*4)*	858	*(69*.*2)*	
Not done	717	*(18*.*5)*	94	*(18*.*8)*	157	*(22*.*5)*	117	*(8*.*2)*	349	*(28*.*1)*	
Unknown	709	*(18*.*3)*	2	*(0*.*4)*	166	*(23*.*7)*	508	*(35*.*5)*	33	*(2*.*7)*	
**Mediastinoscopy**		* *		* *	* *		* *		* *		<0.001
Done	182	*(4*.*7)*	46	*(9*.*2)*	18	*(2*.*6)*	8	*(0*.*6)*	110	*(8*.*9)*	
Not done	2,493	*(64. 4)*	453	*(90*.*4)*	523	*(74*.*8)*	432	*(30*.*1)*	1,085	*(87*.*5)*	
Unknown	1,198	*(30*.*9)*	2	*(0*.*4)*	158	*(22*.*6)*	993	*(69*.*3)*	45	*(3*.*6)*	
**Endobronchial ultrasound**		* *		* *	* *		* *		* *		<0.001
Done	490	*(12*.*7)*	87	*(17*.*4)*	62	*(8*.*9)*	2	*(0*.*1)*	339	*(27*.*3)*	
Not done	2,193	*(56*.*6)*	412	*(82*.*2)*	482	*(68*.*9)*	434	*(30*.*3)*	865	*(69*.*8)*	
Unknown	1,190	*(30*.*7)*	2	*(0*.*4)*	155	*(22*.*2)*	997	*(69*.*6)*	36	*(2*.*9)*	
**Any endoscopy** ^**d**^		* *		* *	* *		* *		* *		<0.001
Done	2,551	*(65*.*9)*	438	*(87*.*4)*	396	*(56*.*7)*	814	*(56*.*8)*	903	*(72*.*8)*	
Not done/unknown	1,322	*(34*.*1)*	63	*(12*.*6)*	303	*(43*.*3)*	619	*(43*.*2)*	337	*(27*.*2)*	
**Combined diagnostic examination**		* *		* *		* *		* *		* *	<0.001
Conventional chest imaging only	89	*(2*.*3)*	4	*(0*.*8)*	7	*(1*.*0)*	60	*(4*.*2)*	18	*(1*.*5)*	
Spiral CT or PET or MRI^e^ + Any endoscopy	2,551	*(65*.*9)*	438	*(87*.*4)*	396	*(56*.*7)*	814	*(56*.*7)*	903	*(72*.*8)*	
Spiral CT or PET or MRI	845	*(21*.*8)*	50	*(10*.*0)*	146	*(20*.*9)*	359	*(25*.*1)*	290	*(23*.*4)*	
Not done/unknown	388	*(10*.*0)*	9	*(1*.*8)*	150	*(21*.*4)*	200	*(14*.*0)*	29	*(2*.*3)*	

^a^ *χ*^2^ tested the between country statistical significance of the differences in the proportions.

^b^ Spiral computed tomography.

^c^ Positron emission tomography.

^d^ Diagnostic endoscopic examinations: bronchoscopy or mediastinoscopy or endobronchial ultrasound.

^e^ Magnetic resonance imaging.

### 2.2. Statistical analysis

To describe variability related to the patient's LC and their characteristics between countries, we used counts and proportions. Differences between countries in the proportions of LC diagnostic examinations were tested with a Chi-square test.

The odds (with 95% confidence intervals (CI) of receiving surgery (for patients with TNM stage I–II at diagnosis) and the odds of receiving chemo- or radiotherapy only (for patients with TNM stage III–IV at diagnosis) or not receiving the treatment, were calculated with multivariable logistic regression models (19) adjusted by age, sex, ICD-O codes for topography, morphology, TNM stage, comorbidity and country. Countries' ORs were based on the differences from the balanced grand mean; the common reference for the areas is therefore their grand mean (20).

Relative survival (RS), i.e., the ratio of the observed to the expected survival in the general underlying population, was calculated by the actuarial method (21). We estimated expected survival by the Ederer II method (22) using CR population life tables stratified by sex, age and year of diagnosis. Country-specific RS figures were standardised to the age structure of the study population.

To assess the impact on five-year risk of death of the variables under study (age and sex, tumour topography, morphology and stage, treatment, comorbidity, smoking status and country of residence), the Relative Excess of Risk of death (RER) five years after diagnosis, with 95% CI, was calculated with generalized linear models, using five-year RS as the dependent variable (23).

Three distinct models were fitted: Model 1) including all countries (4,449 cases) adjusted for all clinical-demographic variables in study (age at diagnosis, sex, topography, morphology, stage at diagnosis, treatment, country); Model 2), adding comorbidity to model 1, including the four countries with data on comorbidity (Belgium, Estonia, Portugal, Spain: 3,720 cases); Model 3) adding to model 1 the adjustment for both comorbidity and smoking, hence including the three countries with data on both covariates (Belgium, Portugal, Spain: 2,214 cases).

Subjects with unknown treatment (153, 3.3%) were excluded from these analyses.

In order to describe the percentage of total between-country variation due to heterogeneity rather than chance, we applied the *I*^2^ statistics to the distribution by country of the estimated RERs and to the Odds of surgical intervention in stage I–II patients or of medical treatments only in stage III–IV patients; the statistical significance threshold of the index was defined at *p* < 0.05 (24, 25).

## 3. Results

### 3.1. Study population characteristics

After excluding 63 cases known only from their death certificate (DCO), we analysed 4,602 LC cases. Table 1 shows the number of cases and the percentages of patients and tumour characteristics by country.

In all countries the percentage of patients aged over 70 was the highest, range 47.6 (Switzerland) to 54.7 (Belgium), except in Portugal, where patients aged 55–69 were the majority (44.7%). The majority of cases were men (75.9%) with similar between country proportions, while Switzerland had the highest percentage of women (40.7%).

In the majority of cases the cancer was located in the lobes, most frequently in the upper lobe (44.3%), while 7.8% were in the main bronchus. Ill-defined or overlapping topographies were 18.4% overall, ranging from 12.9 (Switzerland) to 31.1% (Belgium).

For more than 90% cases the LC diagnosis was microscopically verified (MV), with lower percentages in Estonia (80%) and highest in Belgium (95.6%).

Seventy-eight percent of cases were NSCLC and 16.7% were SCLC. In Portugal there were the highest percentages of NSCLC (81.8%) and the lowest of SCLC (14.3%). In all, NOS morphology cases were 5%, with the highest percentages in Estonia (10.9%) and Switzerland (6.7%).

Fifty-one percent of patients were diagnosed with TNM stage IV; those with stage I or II were 10.8% and 7.4%, respectively. The highest percentages of cases with unknown tumour stage at diagnosis were in Belgium (16.8%) and Switzerland (17%).

Approximately 30% of patients (27.9%) did not receive any anti-cancer treatment or received only palliative care, with the lowest percentage in Switzerland (18.9%). This was 11.3% for stage I–II, 30.5% for stage III–IV and 40.4% for patients with unknown stage at diagnosis.

Surgery, with or without adjuvant treatment (chemotherapy, radiotherapy, or target drugs) was done for 19.9% of patients and 49.0% received only radiotherapy or Chemo- or radiotherapy only, ranging from 33.6 (Estonia) to 61.7 (Belgium).

For patients with stage I–II at diagnosis, the most frequent therapy was surgery (66.5%), ranging from 48.0 (Belgium) to 76.2 (Switzerland); most patients (59.1%) with stage III–IV at diagnosis were given chemo- or radiotherapy only, ranging from 44.9% (Estonia) to 74.1% (Belgium).

Among the countries providing comorbidity data, 42% presented no comorbidity at diagnosis (range 25.7% in Spain to 54.6% in Portugal). Respectively 17.4% and 16.3% patients had low or severe comorbidity. A larger proportion of women than men presented no comorbidity at diagnosis (51% vs. 31%) (data not shown).

Smoking data were available for Belgium, Spain, North Portugal CR and Switzerland, for a total of 2,970 cases. Among these, 38% were current smokers (38.1%), ranging from 33.1 (Belgium) to 48.0 (Spain). A lower percentage of women than men were current or past smokers (39% vs. 57%) and 19% women vs. 3% men were never smokers (data not shown).

### 3.2. Diagnostic work-up

Table 2 lists the numbers of cases and distributions (%) of diagnostic examinations on the lung within three months (after or before) from diagnosis in the four countries with information on diagnostic work-up. Conventional chest imaging (radiography, CT scan or pulmonary stratigraphy) was scheduled for 68.0% of patients (range: 45.1 Portugal to 90.0 Belgium). Seventy-four percent of patients received spiral CT, the most frequent examination in all countries (range: 49.3 Portugal to 94.6 Belgium) and PET was carried out for 35.3% of patients (range: 8% Estonia to 59.5 Belgium); MRI was used for less than 10% in all countries except Portugal (10.5%).

Bronchoscopy was done in 63% cases (range: 53.8 Estonia to 80.8 Belgium); mediastinoscopy was done for 5% (range: 0.6 Portugal to 9.2 Belgium); while in 13% endobronchial ultrasound was done (range: 0.1 Portugal to 27.3 Spain). At least one endoscopic examination was scheduled for 65.9% of the patients, with the highest percentages in Belgium (87.4).

The combination of diagnostic examinations indicated that the majority of patients (2,551, 65.9%) received a complete diagnostic work-up (at least one imaging examination—spiral CT or PET or MRI, plus at least one endoscopic diagnostic examination (bronchoscopy, mediastinoscopy or endobronchial ultrasound).

For 21.8% patients' diagnosis was based on spiral CT or PET or MRI and for 2.3% the diagnosis was based only on conventional chest imaging.

### 3.3. Adhesion to clinical guidelines

Table 3 shows the odds ratios and 95% confidence intervals of undergoing surgery or receiving only chemo- or radiotherapy, by tumour stage group (I–II and III–IV) adjusted by age at diagnosis, sex, topography, morphology, stage, comorbidity and country.

**Table 3:** Adjusted odds ratios (ORs) and 95% confidence intervals (CIs) of surgery for lung cancer patients with tumour stage I–II, or receiving chemo- or radiotherapy only for patients with tumour stage III–IV at diagnosis.

	OR for surgery			OR for chemo- or radiotherapy only		
	Stage I–II (*n* = 691)			Stage III–IV (*n* = 2,885)		
	*N* of cases	OR	95% CI	N of cases	OR	95% CI
**Age at diagnosis**						
15–54	55	ref	299	ref
55–69	220	1.16	0.59–2.27	799	0.74	0.56–0.96
≥70	170	0.51	0.26–0.99	599	0.34	0.26–0.44
**Sex**						
Men	340	ref	1,331	ref
Women	105	1.60	0.99–2.59	366	0.98	0.80–1.21
**Topography**						
Lower, middle/upper lobe	417	ref	1,243	ref
Main Bronchus	6	0.25	0.08–0.82	145	0.83	0.62–1.11
Site Not Otherwise Specified	22	0.38	0.20–0.74	309	0.79	0.64–0.98
**Morphology**						
Non-small-cell carcinoma	383	ref	1,296	ref
Small-cell carcinoma	54	1.12	0.58–1.87	314	1.26	1.00–1.58
Not Otherwise Specified	8	0.07	0.03–0.15	87	0.20	0.15–0.26
**TNM Stage grouping**						
I	301	ref	–	–
II	144	0.42	0.29–0.60	–	–
III	–		491		ref
IV	–		1,206	1.09	0.91–1.30	
**Comorbidity** ^a^						
Absent (0 points)	167	ref			
1–2 points	195	0.83	0.54–1.27	–	–
> 2 points	80	0.55	0.33–0.93	–	–
No. (0–1 point)				1,189	ref
≥2 points				484	0.82	0.69–0.98
Unknown	3	2.10	0.28–15.59	14	0.26	0.13–0.54
**Country** ^b^						
Belgium	48	0.62	0.42–0.91	234	1.92	1.55–2.38
Estonia	109	1.82	1.28–2.60	207	0.63	0.53–0.75
Portugal	142	0.69	0.52–0.93	696	0.86	0.75–0.98
Spain	146	1.28	0.94–1.76	560	0.97	0.84–1.12

^a^ Comorbidity was classified as 0; 1–2; > 2, unknown, in the model analyzing OR of receiving surgery; as 0–1, ≥2, unknown in the model analyzing OR of receiving medical treatment only.

^b^ Reference: mean of the four countries. Switzerland was not included in these models, as comorbidity data were not available.

Surgery was scheduled for 20% of total patients, but considering patients who had tumour stage I–II at diagnosis, for whom surgery is indicated, this percentage rose to 66% overall. Approximately 30% of patients had no specific cancer treatment, with lower percentages in Belgium and Switzerland.

Among the 281 patients with stage I–II who did not have surgery, medical contraindications were reported for 70 cases, patient's refusal for 21, but for 190 cases the reasons were unknown or unspecified.

For stages I–II, the adjusted OR of surgery decreased with advancing age; it was lower for cancer located in the main bronchus (OR 0.25, CI, 0.08–0.82) and not specified site (OR 0.38, CI, 0.20–0.74) than in other subsites; for TNM stage II (OR 0.42, CI, 0.29–0.60) than stage I and for patients with severe comorbidity (OR 0.55, CI, 0.33–0.93) than those with no comorbidity. The adjusted OR for surgery was higher than the mean in Estonia (OR1.82, CI, 1.28–2.60) and lower than the mean in Belgium (OR 0.62, CI, 0.42–0.91) and Portugal (OR 0.69, CI, 0.52–0.93).

Considering patients with tumour stages III–IV, the OR of receiving chemo- or radiotherapy decreased significantly with age; it was lower for cancers of ill-defined topography (OR 0.79, CI, 0.64–0.98) than other subsites. With reference to NSCLC, the OR was lower for NOS morphology (OR 0.20, CI, 0.15–0.26) and higher for SCLC (OR 1.26, CI, 1.00–1.58). The adjusted OR was higher than the mean in Belgium (OR 1.92, CI, 1.55–2.38) and lower in Estonia (OR 0.63, CI, 0.53–0.75) and Portugal (OR 0.86, CI, 0.75–0.98).

The *I*^2^ statistics for ORs of surgical intervention in stage I–II patients was 97.44% and 99.70% for ORs of chemo or radiotherapy in stage III–IV.

### 3.4. Survival

**Figure 1:**
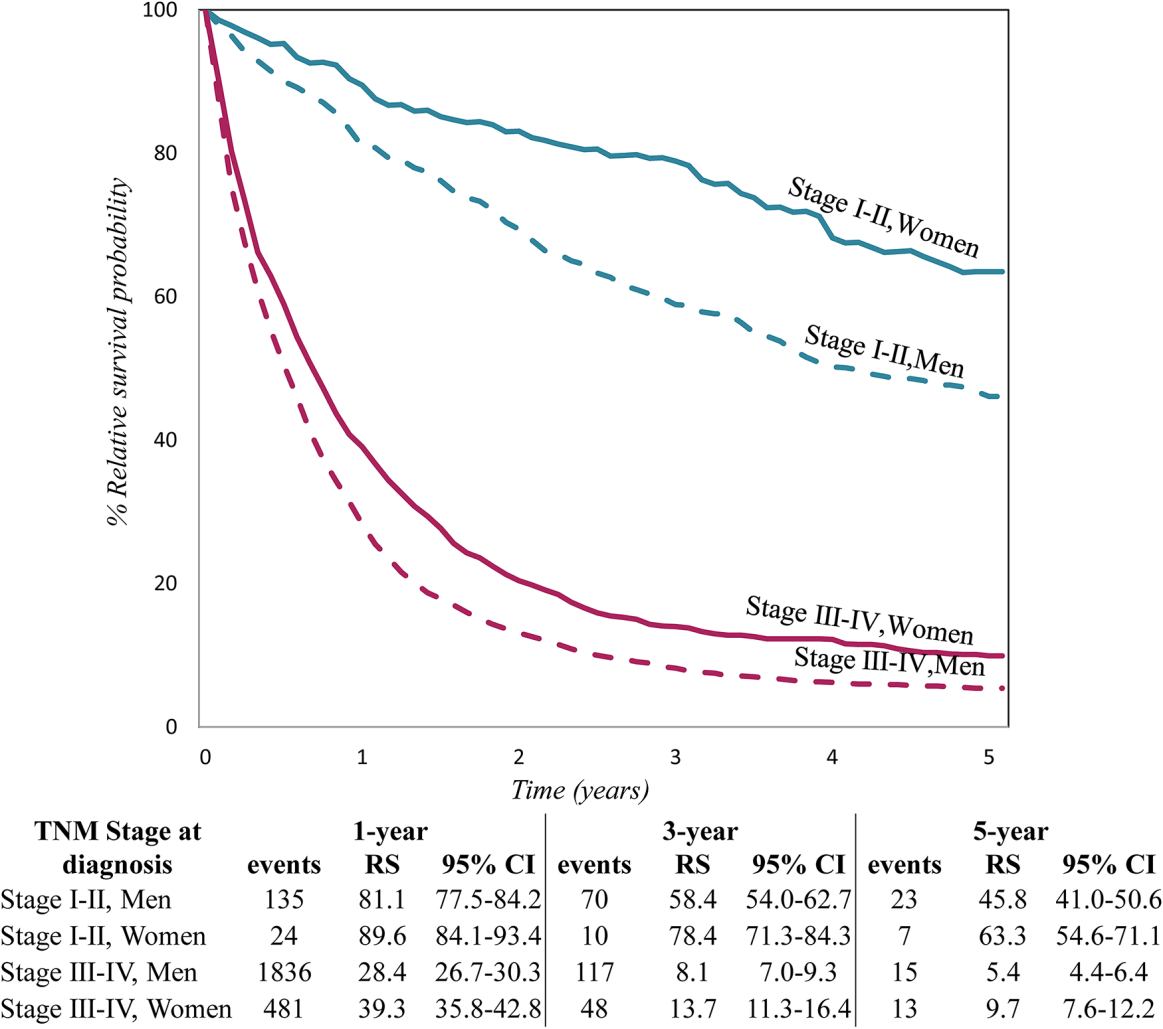
Relative survival by TNM stage at diagnosis and sex, for lung cancer patients diagnosed in 2009–2013 in five European countries.

Figure 1 shows relative survival for patients with tumour stages I–II and III–IV, by sex at 1, and 5 years after diagnosis, with 95% CI. In all tumour stage groups, women had longer survival than men but in stage III–IV the difference by sex decreased over time.

**Figure 2:**
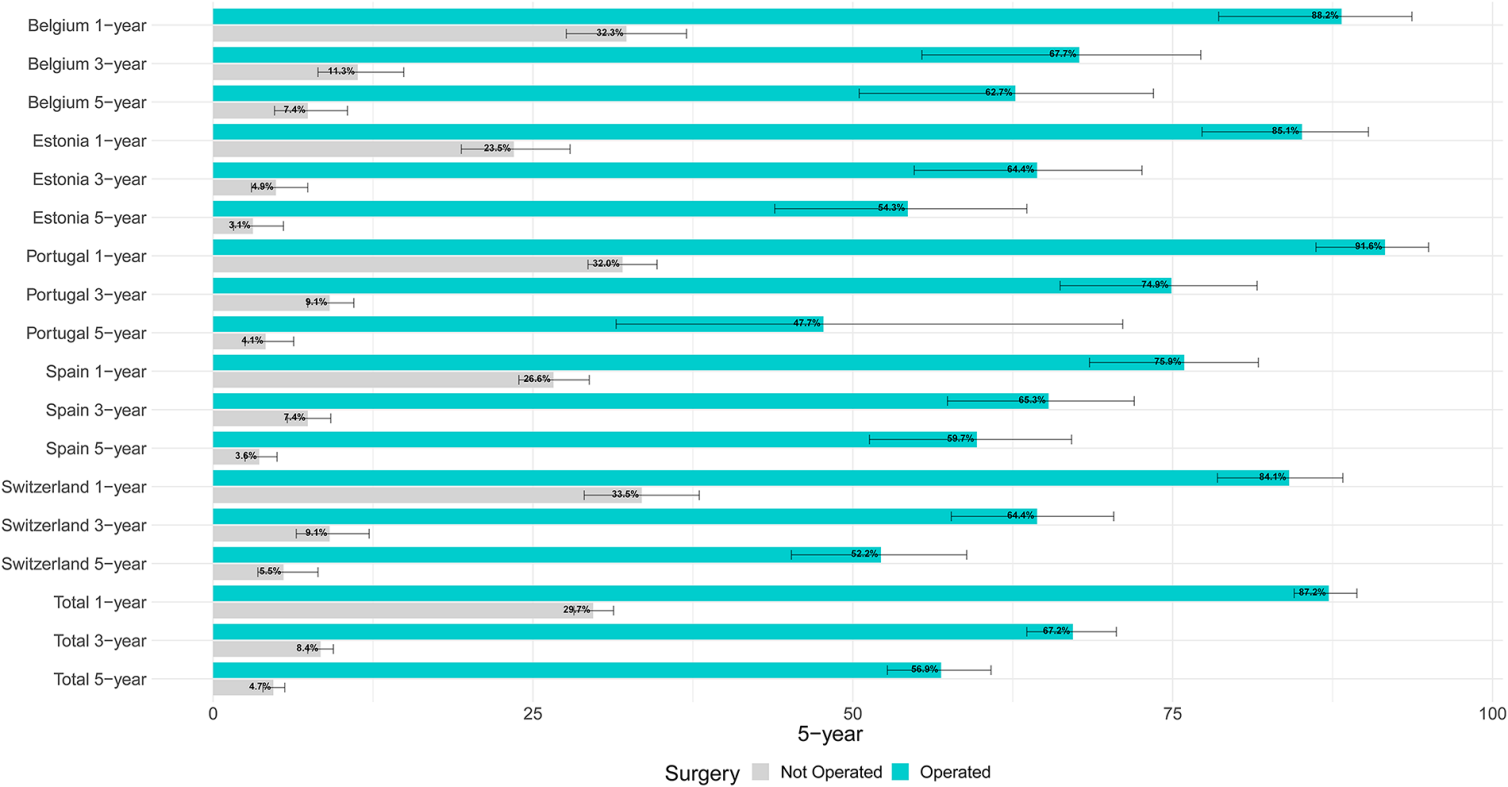
Age-adjusted relative survival by country and treatment for lung cancer patients diagnosed in 2009–2013 in five European countries.

Figure 2 shows age-standardised relative survival (RS) with 95% confidence intervals by country for operated and not operated cases. In all, 87.2% of operated cases were alive one year after diagnosis, ranging from 84.1% in Switzerland to 91.6% in Portugal; three years after diagnosis the overall RS had fallen to 67.2%. The 5-year RS of total operated cases was 56.9, ranging from to 47.7 in Portugal to 62.7 in Belgium. For non-operated cases, the overall 1-, 3- and 5-year RS figures were respectively 29.7, 8.4, 4.7, with little across-country differences. Table 4 shows the results of the multivariable analysis to assess the impact of the factors under study on five-year risk of death.

**Table 4:** Adjusted five-year relative excess of risk of death (RER) with 95% confidence intervals (CI) for lung cancer patients diagnosed in 2009–2013 in five European countries.

Variables		Mod 1 (all countries, *n* = 4,449)		Mod 2 (Belgium, Spain, Estonia, North Portugal, *n* = 3,720)		Mod 3 (Belgium, Spain, North Portugal, *n* = 2,214)	
		RER	95% CI	RER	95% CI	RER	95% CI
**Age at diagnosis (years)**	15–54	ref	ref	ref
	55–69	1.04	0.94–1.17	0.98	0.87–1.11	1.04	0.89–1.22
	≥70	1.14	1.02–1.28	1.09	0.96–1.23	1.16	0.98–1.37
**Sex**	Men	ref	ref	ref			
	Women	0.78	0.72–0.86	0.78	0.71–0.86	0.88	0.77–1.01
**Topography**	Lower, middle or upper lobe	ref	ref	ref
	Main Bronchus	1.37	1.21–1.54	1.51	1.32–1.73	1.54	1.32–1.80
	Not Otherwise Specified	1.14	1.04–1.25	1.15	1.05–1.27	1.12	0.98–1.29
**Morphology**	Non-small-cell carcinoma	ref	ref	ref
	Small-cell carcinoma	0.97	0.88–1.07	0.98	088–1.09	0.97	0.85–1.11
	Not Otherwise Specified	1.17	1.05–1.30	1.17	1.04–1.31	1.35	1.16–1.57
**TNM stage grouping at diagnosis**	I	ref	ref	ref
	II	1.87	1.48–2.35	1.92	1.49–2.47	1.79	1.30–2.45
	III	2.60	2.15–3.14	2.65	2.16–3.27	2.17	1.69–2.80
	IV	5.13	4.27–6.18	5.19	4.24–6.36	4.49	3.50–5.76
	Unknown	2.63	2.12–3.27	2.98	2.32–3.83	3.06	2.21–4.25
**Treatment**	No anticancer treatment	ref	ref	ref
	Surgery	0.13	0.11–0.15	0.12	0.10–0.15	0.12	0.09–0.15
	Medical treatment only	0.37	0.34–0.40	0.37	0.33–0.40	0.37	0.33–0.42
**Comorbidity**	No (0 point)			ref	ref
	Yes (>1 point)			1.09	1.01–1.18	1.21	1.09–1.35
	Unknown			0.92	0.70–1.20	1.06	0.78–1.43
**Smoking status**	Current					ref
	Never					0.68	0.57–0.81
	Previous					0.95	0.85–1.06
	Unknown					1.05	0.90–1.23
**Country** ^**a**^	Belgium	0.97	0.89–1.06	0.96	0.88–1.05	0.96	0.88–1.05
	Estonia	1.20	1.10–1.30	1.16	1.07–1.26	-	
	Portugal	0.88	0.82–0.93	0.87	0.82–0.92	0.97	0.89–1.05
	Spain	1.07	1.00–1.14	1.03	0.97–1.10	1.07	1.00–1.15
	Switzerland	0.91	0.84–0.99	–		–	

Mod 1 is adjusted for age, sex, topography, morphology, TNM stage grouping at diagnosis, treatment, country; Mod 2 is adjusted for age, sex, topography, morphology, TNM stage grouping at diagnosis, comorbidity, treatment, country;Mod 3 adjusted for age, sex, topography, morphology, TNM stage grouping at diagnosis, comorbidity, smoking status, treatment, country.

^a^ Reference: mean of the five countries. Estonia was not included in model 3 as smoking data were not available; Switzerland was not included in model 2 and 3, as comorbidity and smoking data were not available.

Considering model 1, the adjusted five-year RER increased with age at diagnosis, significantly for patients aged 70 and over (RER 1.14, CI, 1.02–1.28). The RER was lower for women (RER 0.78, CI, 0.72–0.86) than men. It was higher for cancers in the main bronchus (RER 1.37, CI, 1.21–1.54) than other subsites, for NOS morphology (RER 1.17, CI, 1.05–1.30) than NSCLC. The RER rose significantly with worse stage at diagnosis.

Compared to patients receiving no anti-cancer treatment, those treated surgically had the lowest mortality (RER 0.13, CI, 0.11–0.15), but those receiving chemo- or radiotherapy only also had lower than reference RER (RER 0.37, CI, 0.34–0.40). The fully adjusted five-year RER was higher than the mean in Estonia (RER 1.20, CI, 1.10–1.30) and lower in Portugal (RER 0.88, CI, 0.82–0.93) and Switzerland (RER 0.91, CI, 0.84–0.99).

The analyses restricted to the subsets of CRs with information on comorbidity (model 2) and both comorbidity and smoking (model 3) provided RERs and CI very similar to those of model 1 for age, sex, topography, morphology, stage, treatment and country.

In addition, these models indicated that patients with any comorbidity at diagnosis had significantly higher RER than those with no comorbidity (RER 1.09, CI, 1.01–1.18 in model 2; RER 1.21, CI, 1.09–1.35 in model 3). Model 3 also showed that the adjusted RER of never smokers was lower than that of current smokers (RER 0.68, CI, 0.57–0.81).

A larger proportion of women than men presented no comorbidity at diagnosis (51% vs. 31%), 29% vs. 36% was current, 17% vs. 45% was past smoker and 37% vs. 5% was never smoker). Adjustment for these factors and for clinical-pathological characteristics in the multivariable models confirmed the independent effects of sex on survival.

The *I*^2^ statistics for the distribution in the RERs across countries varied from 0.00 to 0.01, indicating small between country heterogeneity in the RER (i.e., heterogeneity is mostly due to the other model covariates).

## 4. Discussion

Consistently with previous studies (4–7). In our study approximately 20% (or less) LC patients were diagnosed with early stage tumours potentially amenable to curative surgery. More than half, however, had advanced tumours, a condition that precludes the possibility of curative treatment. These results point to the need to improve access to timely diagnosis.

We uncovered geographical differences in adhering to the clinical guidelines under study (surgery in stage I–II, medical treatment in stage II–IV); the *I*^2^ high values in the ORs models indicate these variations are attributable to real heterogeneity across countries.

Although LC survival was uniformly low in all countries, surgery was independently and significantly associated with lower mortality and compared to patients receiving no anticancer treatment, chemo- or radiotherapy also offered a significant protective effect.

Our protocol did not investigate the intent to treat; however, for of 82% operated patients, radical surgery was possible and was highly protective of mortality risk, so we maintain that surgery was scheduled with curative intent. In addition to the factors captured by our study, survival of operated patients may also be related with the expertise and training of the surgeons, the number of cases performed by individual surgeons or the case load of the hospitals within the registry. Likewise, difference in experience and training among medical and radio oncologists could account for difference in outcomes.

The geographical differences in LC survival evident in our univariate analyses, and also reported in previous studies (3, 4), were attenuated by adjustment for clinical characteristics (morphology, topography), sex, age, treatment, comorbidity or smoking. In fact, the low values for *I*^2^ in the RER distribution across countries, suggest the clinical pathological covariates included in the models are main determinants of survival, while variations by country are less important.

Accurate diagnostic investigation is essential for guiding therapy and for selecting patients who can benefit from surgery. The selection of patients for surgery affects its outcome: in countries with higher proportions of surgically treated patients, more frail patients may have been treated and the outcomes therefore might be worse.

In our study more than 65% of patients received imaging combined with an endoscopy. In Belgium, where this percentage was the highest, the proportion of stage I–II patients treated surgically was lower and the proportion of patients receiving medical treatment (chemo- or radiotherapy) was higher than in the other countries, and five-year relative survival of the operated cases was among the highest in the countries analysed. By contrast, in Estonia where the percentage of extensive diagnostic work-up was lower than average, the odds of surgery for stage I–II cases was higher than average and survival lower.

Although in Estonia the probability of surgical treatment for localised lung cancer increased markedly from the late 1990s to the 2010s, even for SCLC, correlating with increasing survival rates (5), in the present study survival was lower than in other countries, for both surgically and medically treated LC patients. In Estonia, the limited availability of radiotherapy, particularly stereotactic body radiation therapy (SBRT), could have contributed to the worse survival of patients who were not operated. Estonia was among the countries with the lowest availability of radiotherapy equipment in Europe until 2012 (26). SBRT was not available in 2011, molecular targeted therapies for NSCLC were reimbursed from 2010, and immunotherapy became available after 2014.

Data on the diagnostic work-up was not available for Switzerland, where five-year survival of operated cases was lower than in other countries, and the corresponding figure for non-operated cases was among the highest.

These findings confirm the importance of accurate diagnostic profiling to select patients who will benefit from surgery.

In line with other studies reporting that the frequency of surgery decreased with age (5, 27), we found that the odds of undergoing surgery for stage I–II LC patients did in fact decrease with age, stage, comorbidity and location of the cancer in the main bronchus. The worse survival with greater age at diagnosis explained the decreases in the frequency of surgery with later age.

Although surgery is mainly recommended for early stage NSCLC (9), we found the odds of receiving surgery in stage I–II did not differ between SCLC and NSCLC and the ratio was reduced only for ill-defined histotypes (NOS). Coherently with clinical guidelines (8), among stage III–IV cases SCLC were more likely than NSCLC to receive medical treatment (chemo- or radiotherapy).

Our results are in line with studies suggesting resection might be indicated also for very early SCLC (28–30) and with population-based studies reporting that from 1% to 25.1% SCLC received surgery in the UK (31). According to many studies, SCLC have a worse prognosis than NSCLC (3, 5). In our study three-year survival of SCLC cases was 27.9%, vs. 33.4% for NSCLC, but once all the factors in the multivariable model were adjusted, only patients with ill-defined histology (NOS) had higher than the reference RER. This is in line with reports that the survival of operated SCLC can be comparable to that of NSCLC patients (29).

Probably on account of the problematic anatomic suitability for resection and reconstruction, and the technical complexity of surgery for tumours of the main bronchus and carina (32, 33), only 7 of the 358 patients with cancer in the main bronchus were treated surgically, while 53% of total surgeries involved lobectomy to resect peripheral cancers in the lobes. Coherently with the low frequency of surgery, location in the main bronchus carried higher RER than other sites, while there were no RER differences amongst lobectomy, segmentectomy or pneumectomy. We have no data on minimally invasive surgery, which has advanced rapidly, making surgery an acceptable treatment option even for elderly and frail patients and those with comorbidities. The use of thoracoscopic surgery may have differed between countries at the time of data collection.

Using data of six CRs in the four countries that provided less than 12% of cases with unknown comorbidity, we found severe comorbidity (CCI >2) was associated with omission of surgery for stage I–II cancers, as well as with the odds of receiving chemo- or radiotherapy for stage III–IV patients. We cannot exclude that for some cases for whom CCI was coded as “no comorbidity” (CCI = 0), the relevant information was actually unknown, this bias could have led to underestimating the effect of comorbidity on RERs and ORs.

Our findings are in line with reports suggesting that between 24% and 70% of cancer patients with comorbidity are not treated according to guidelines (34). The probability of successful surgery is reduced with advanced age and the presence of comorbidities, also due to the expected higher incidence of postoperative complications (18).

In accordance with other studies (14, 35), in the multivariable analysis restricted to the CRs providing the relevant data, comorbidity and smoking habit were independently and positively associated with RER.

Coherently with other studies (14), LC patients who are current smokers had a higher risk of death than non-smokers. The most frequent comorbidities in our study population were COPD and CVD, both related to smoking, operatory risk and mortality. Severity of comorbidity was also associated to mortality (data not shown).

While in many western countries LC incidence is decreasing for men, an increase over the recent decades is evident for women (1, 36), so the numbers of cases among women can be expected to rise in the next few years, with specific health needs. Identifying predictive prognostic factors can help tailor sex-specific disease management (37–40).

The survival advantage of women also found in other studies has been attributed to earlier tumour stage at diagnosis, better health behaviour, lower comorbidity (41), all factors related to the possibility of curative treatment. Contrary to a report from England (31), in our study surgery did not differ by sex; nor did the stage distribution (stage I–II was 17% in women and 18% in men; stage III–IV was 69% and 72%, respectively). In each stage category survival was higher for women than men (Figure 1).

Although a larger proportion of women than men presented no comorbidity at diagnosis and were non-smokers, adjustment for these factors and for clinical-pathological characteristics in the multivariable models confirmed the independent effects of sex on survival.

### 4.1. Strengths and limits

A major strength of our study consists in the large number of cases and in its population basis with recruitment of all incident cases in the study period or registry sub-area, with collection of *ad hoc* data additional to those routinely available to cancer registries, using a common protocol and analysis methods. Thus, our study provides information concerning standard care and treatment effectiveness in everyday clinical practice without any patient or outcome selection, and is therefore representative of what happens in real-life.

A limit of the study is its retrospective observational design, with variables abstracted from clinical records, which are not structured nor standardised across hospitals and countries. Hence, a part of differences in care and outcomes found by our study may depend on data availability and clinicians' attitudes to reporting.

Epidermal growth factor receptor (EGFR) testing was done in 6% of NSCLC, with small across-registry differences. In a similar study period, other population data report 45% of cases tested (42), so we have to consider the percentage found in our study unreliable. However, clinical guidelines (43) concluded that EGFR-targeted agents were not associated with better survival in the subgroup with EGFR-mutated LC (43).

## 5. Conclusions

Although the prognosis for LC is uniformly poor in all countries, the survival benefit of diagnosis at early stages, allowing curative surgery, was confirmed by multivariable analyses adjusted by clinic, pathological and demographic factors, comorbidity and smoking. Our findings support the importance of accurate diagnostic investigation for selecting patients who can benefit from surgery, and of screening for subjects at risk, to detect lung cancer at a very early stage when curative treatment is applicable.

Early diagnosis should be increased through screening for subjects at risk and public health campaigns on awareness of LC symptoms; adhesion to standard care should be incremented by funding better equipment across the EU, and better training of health personnel.

## Data Availability

The raw data supporting the conclusions of this article will be made available by the authors, without undue reservation.
