# Supplementary material for: Patterns of care and survival for lung cancer: Results of the European population-based high-resolution study
Source: Front Epidemiol. 2023 Mar 3;3:1109853. doi: 10.3389/fepid.2023.1109853 (PMC10910949; doi:10.3389/fepid.2023.1109853)
Supplement: Supplementary file 1 [file Table1.docx]

**Table S1. ICDO-3-morphologyical codes for tumors with microscopic basis of diagnosis.**

| **Morphological subgroup** | **ICDO-3 morphological code** |
| --- | --- |
| Unspecified carcinoma (NOS) | 8000; 8001; 8010; 8020; 8021; 8022; 8031 |
| Squamous carcinoma , Non-small-cell lung cancer, Other, Large cell carcinoma, Adenocarcinoma (NSCLC) | 8012; 8033; 8046; 8052; 8070; 8071; 8072; 8073; 8074; 8076; 8082; 8083; 8084; 8123; 8140; 8200; 8211; 8230; 8250, 8251, 8252, 8253; 8255; 8260; 8310; 8323; 8430; 8480; 8481; 8490; 8550; 8560; 8570;8800; 8815; 8980; 9140; 9220; 9530 |
| Small-cell lung cancer, Neuroendocrine carcinoma (SCLC) | 8013; 8041; 8042; 8043; 8044; 8045; 8240; 8244; 8246; 8249; 8574; |
