# Supplementary material for: Patterns of care and survival for lung cancer: Results of the European population-based high-resolution study
Source: Front Epidemiol. 2023 Mar 3;3:1109853. doi: 10.3389/fepid.2023.1109853 (PMC10910949; doi:10.3389/fepid.2023.1109853)
Supplement: Supplementary file 2 [file Table2.docx]

**Supplementary Table 2 - Total number of lung cancer cases submitted for inclusion in the HR study by country and registry, with criteria for selection of cases.**

| **Country** | **Cancer Registry** | **Number of cases** | **Criteria for selection** |
| --- | --- | --- | --- |
| Belgium | National | 501 | All incident cases diagnosed between March 1st and March 14th 2011 |
| Estonia | Estonia National | 699 | All incident cases in 2011 |
| Portugal | Northern Portugal | 500 | At least 500 LC cases randomly sampled from the incident cases diagnosed in the last years with complete incidence (i.e., 2009-13 |
| Portugal | Southern Portugal | 933 | All incident cases in 2012 |
| Spain | Girona | 489 | All incident cases in the province of Girona in 2009-2012 |
| Spain | Granada | 751 | All incident cases in 2011 |
| Switzerland | Geneva | 729 | All incident cases in 2011-2013 |
